# Patients’ and Clinicians’ Perceptions of the Clinical Utility of Predictive Risk Models for Chemotherapy-Related Symptom Management: Qualitative Exploration Using Focus Groups and Interviews

**DOI:** 10.2196/49309

**Published:** 2024-06-20

**Authors:** Morven Miller, Lisa McCann, Liane Lewis, Christine Miaskowski, Emma Ream, Andrew Darley, Jenny Harris, Grigorios Kotronoulas, Geir V Berg, Simone Lubowitzki, Jo Armes, Elizabeth Patiraki, Eileen Furlong, Patricia Fox, Alexander Gaiger, Antonella Cardone, Dawn Orr, Adrian Flowerday, Stylianos Katsaragakis, Simon Skene, Margaret Moore, Paul McCrone, Nicosha De Souza, Peter T Donnan, Roma Maguire

**Affiliations:** 1 Computer & Information Sciences University of Strathclyde Glasgow United Kingdom; 2 Johnson and Johnson Medical Norderstedt Germany; 3 University of California San Francisco, CA United States; 4 School of Health Sciences University of Surrey Guildford United Kingdom; 5 School of Medicine University College Dublin Dublin Ireland; 6 School of Medicine, Dentistry and Nursing University of Glasgow Glasgow United Kingdom; 7 Department of Health Sciences Norwegian University of Science and Technology Gjøvik Norway; 8 Department of Internal Medicine 1 Division of Hematology and Hemostaseology Medical University of Vienna Vienna Austria; 9 School of Health Sciences National and Kapodistrian University of Athens Athens Greece; 10 School of Nursing, Midwifery and Health Systems University College Dublin Dublin Ireland; 11 European Cancer Patient Coalition Brussels Belgium; 12 NHS 24 Glasgow United Kingdom; 13 Docobo Limited Leatherhead United Kingdom; 14 Surrey Clinical Trials Unit University of Surrey Guildford United Kingdom; 15 Department of Health Services and Population Research Institute of Psychiatry Psychology and Neuroscience King’s College London London United Kingdom; 16 Population Health and Genomics Medical School University of Dundee Dundee United Kingdom

**Keywords:** chemotherapy, digital health, education, predictive risk models, qualitative research methods, symptoms, symptom cluster, tailored information

## Abstract

**Background:**

Interest in the application of predictive risk models (PRMs) in health care to identify people most likely to experience disease and treatment-related complications is increasing. In cancer care, these techniques are focused primarily on the prediction of survival or life-threatening toxicities (eg, febrile neutropenia). Fewer studies focus on the use of PRMs for symptoms or supportive care needs. The application of PRMs to chemotherapy-related symptoms (CRS) would enable earlier identification and initiation of prompt, personalized, and tailored interventions. While some PRMs exist for CRS, few were translated into clinical practice, and human factors associated with their use were not reported.

**Objective:**

We aim to explore patients’ and clinicians’ perspectives of the utility and real-world application of PRMs to improve the management of CRS.

**Methods:**

Focus groups (N=10) and interviews (N=5) were conducted with patients (N=28) and clinicians (N=26) across 5 European countries. Interactions were audio-recorded, transcribed verbatim, and analyzed thematically.

**Results:**

Both clinicians and patients recognized the value of having individualized risk predictions for CRS and appreciated how this type of information would facilitate the provision of tailored preventative treatments or supportive care interactions. However, cautious and skeptical attitudes toward the use of PRMs in clinical care were noted by both groups, particularly in relationship to the uncertainty regarding how the information would be generated. Visualization and presentation of PRM information in a usable and useful format for both patients and clinicians was identified as a challenge to their successful implementation in clinical care.

**Conclusions:**

Findings from this study provide information on clinicians’ and patients’ perspectives on the clinical use of PRMs for the management of CRS. These international perspectives are important because they provide insight into the risks and benefits of using PRMs to evaluate CRS. In addition, they highlight the need to find ways to more effectively present and use this information in clinical practice. Further research that explores the best ways to incorporate this type of information while maintaining the human side of care is warranted.

**Trial Registration:**

ClinicalTrials.gov NCT02356081; https://clinicaltrials.gov/study/NCT02356081

## Introduction

### Background

Over 19 million people worldwide were diagnosed with cancer in 2020 [1]. By 2040, the global burden is expected to grow to 29.4 million new cancer cases annually, which reflects the growth and aging of the global population [2]. While chemotherapy is one of the most effective cancer treatments [3-5], some patients experience severe and potentially life-threatening symptoms [6-8]. Poor symptom management can result in symptom escalation [9,10], poor adherence to treatment [11,12], reduced health-related quality of life (QoL) [13,14], and treatment-related mortality [9,15]. The value of early detection and prompt symptom management cannot be underestimated given that decreases in symptom severity and rapid resolution are associated with improved patient experiences and treatment outcomes. Moreover, symptoms can persist beyond treatment into survivorship [16], increase the long-term burden of cancer on individuals and their families [6,17,18], and generate significant costs to the health care system and society [19].

### Symptom Management and Information Provision

Accurate prediction of patients who are most likely to experience high levels of symptom burden is clinically challenging [20]. These challenges relate to variability in patients’ demographic (eg, age and gender), clinical (eg, comorbidities), genetic, and socioeconomic characteristics that may be key moderators of patients’ symptom experience, and psychosocial adjustment to cancer treatment [18,21,22].

Traditional approaches to managing symptoms in patients receiving chemotherapy on an outpatient basis rely on patients’ recollection when they attend for subsequent treatments. However, approaches that depend on patients’ recollection are subject to both recall bias and underreporting [23]. Alternatively, people present to acute oncology services with severe symptoms. The ideal scenario would be to predict, pre-empt and, when possible, treat chemotherapy-related symptoms (CRS) prophylactically in addition to effective self-care.

### Prediction of Future Symptom Experiences

During the last decade, enhanced computing power and the availability of large amounts of data have prompted the practical use of artificial intelligence (AI) in health care [24]. AI has the potential to transform health care practices with its increasing ability to translate the uncertainty and complexity of data into actionable clinical decisions [25]. Predictive models have been used to identify patients who are most likely to experience specific disease and treatment-related events [26,27].

One of the first studies to explore the use of predictive risk modeling (PRM) in supportive care developed and tested a symptom risk modeling tool for patients with breast cancer undergoing adjuvant chemotherapy and demonstrated a high level of accuracy in predicting 4 out of 6 symptoms [28]. Recent studies advanced the state of the art by employing person-centered analytical approaches to gain insights into the complex nature of co-occurring symptoms and symptom clusters and the identification of subgroups of patients with distinct symptom profiles [20,29,30]. These findings highlight some of the potential that risk prediction can play in the delivery of person-centered care through the identification of high-risk patients with multiple co-occurring symptoms.

While an evidence base is emerging around the development of PRMs for supportive cancer care, information about clinicians’ and patients’ perspectives on their implementation, utility, and real-world application is limited. This significant gap in the literature warrants evaluation [25,31,32] so that PRMs can be implemented in ways that will improve the quality of care and patient outcomes [33].

### Aim of This Study

This paper reports on an exploratory secondary objective from a larger program of work Electronic Symptom Management Using the Advanced Symptom Management System (eSMART; ClinicalTrials.gov NCT02356081). The primary study was a randomized controlled trial (RCT) that evaluated the effects of real-time remote monitoring on symptom burden, QoL, supportive care needs, anxiety, self-efficacy, and work limitations experienced by patients with cancer receiving chemotherapy [34-37]. eSMART was funded through the European Union’s Seventh Framework Programme for research, technological development, and demonstration (602289EU) and was conducted across 12 clinical sites within 5 European countries (ie, Austria, Greece, Republic of Ireland, Norway, and the United Kingdom). The purpose of the current analysis was to explore patients’ and clinicians’ perceptions of the utility and real-world application of PRMs for CRS.

## Methods

### Participants and Setting

Purposive groups of patients and clinicians were recruited from 12 clinical sites in Austria, Greece, Ireland, Norway, and the United Kingdom. Participants (ie, patients and clinicians) were not required to have participated in the RCT component of eSMART. Given that both patients’ and clinicians’ perspectives were equally important, adequate representation from both groups was sought.

Potential participants were purposively identified and recruited by members of the local health care team to have a sample that was as varied as possible per age, gender, diagnoses (for patients), and clinical roles (for clinicians) to capture a wide range of viewpoints. Patients were eligible if they were diagnosed with breast or colorectal cancer or Hodgkin or non-Hodgkin lymphoma and had received chemotherapy within the past year*.* Clinicians were eligible if they were members of clinical teams involved in the provision of supportive care for patients with breast, colorectal, or any hematological cancer receiving chemotherapy.

### Ethical Considerations

This study was approved by NHS Lothian South East Scotland Research Ethics Committee 02 (14/SS/1062) and sponsored by the University of Strathclyde. This study received ethical approval from each of the study’s sites. All participants provided written informed consent.

### Data Collection

**Figure 1:**
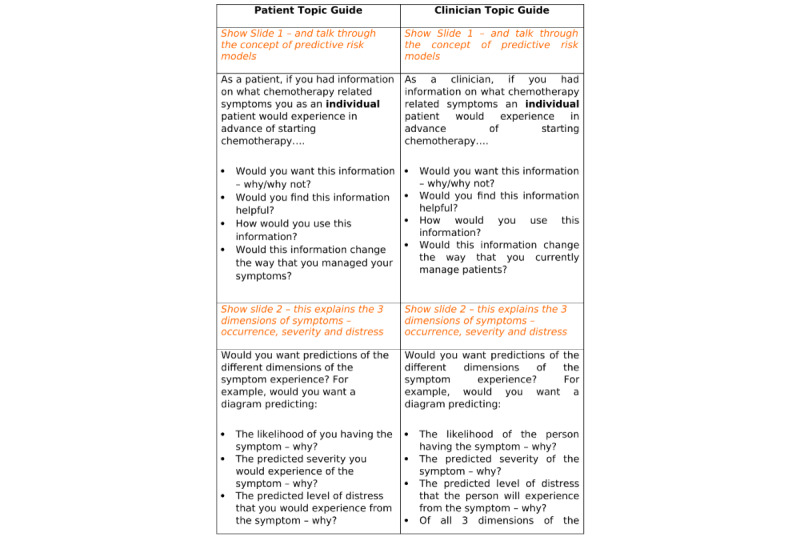
Topic Guide for Clinicians and Patients.

**Figure 2:**
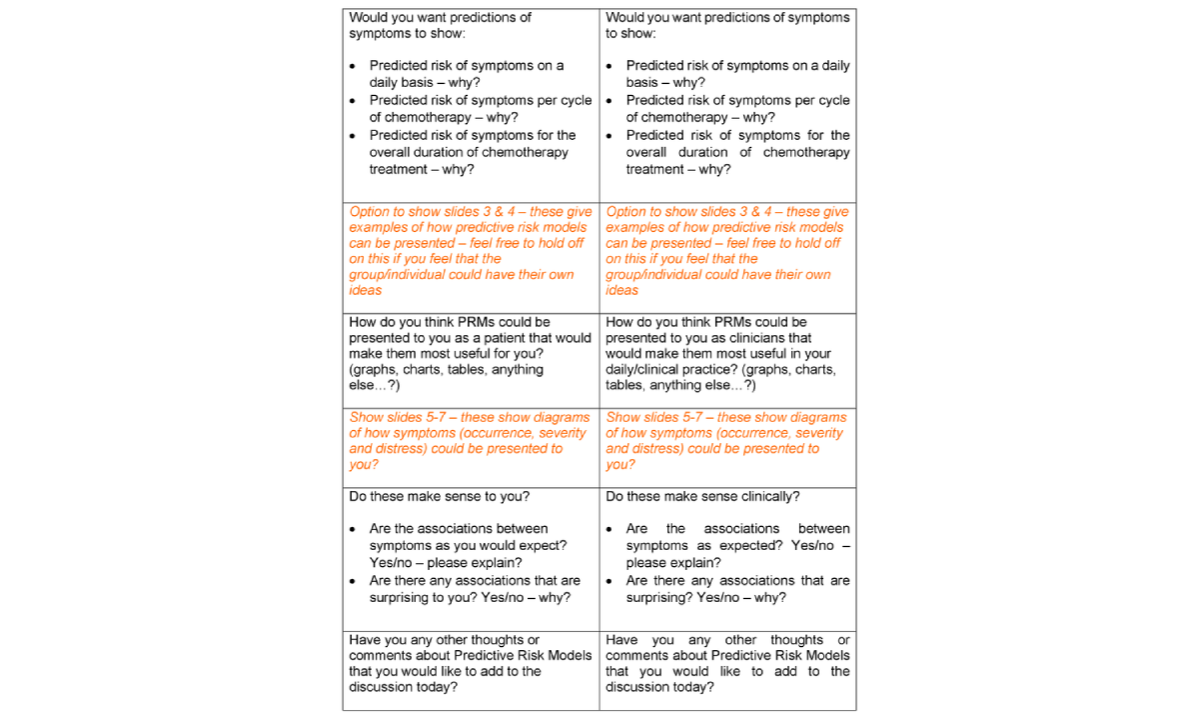
Topic Guides for Clinicians and Patients.

**Figure 3:**
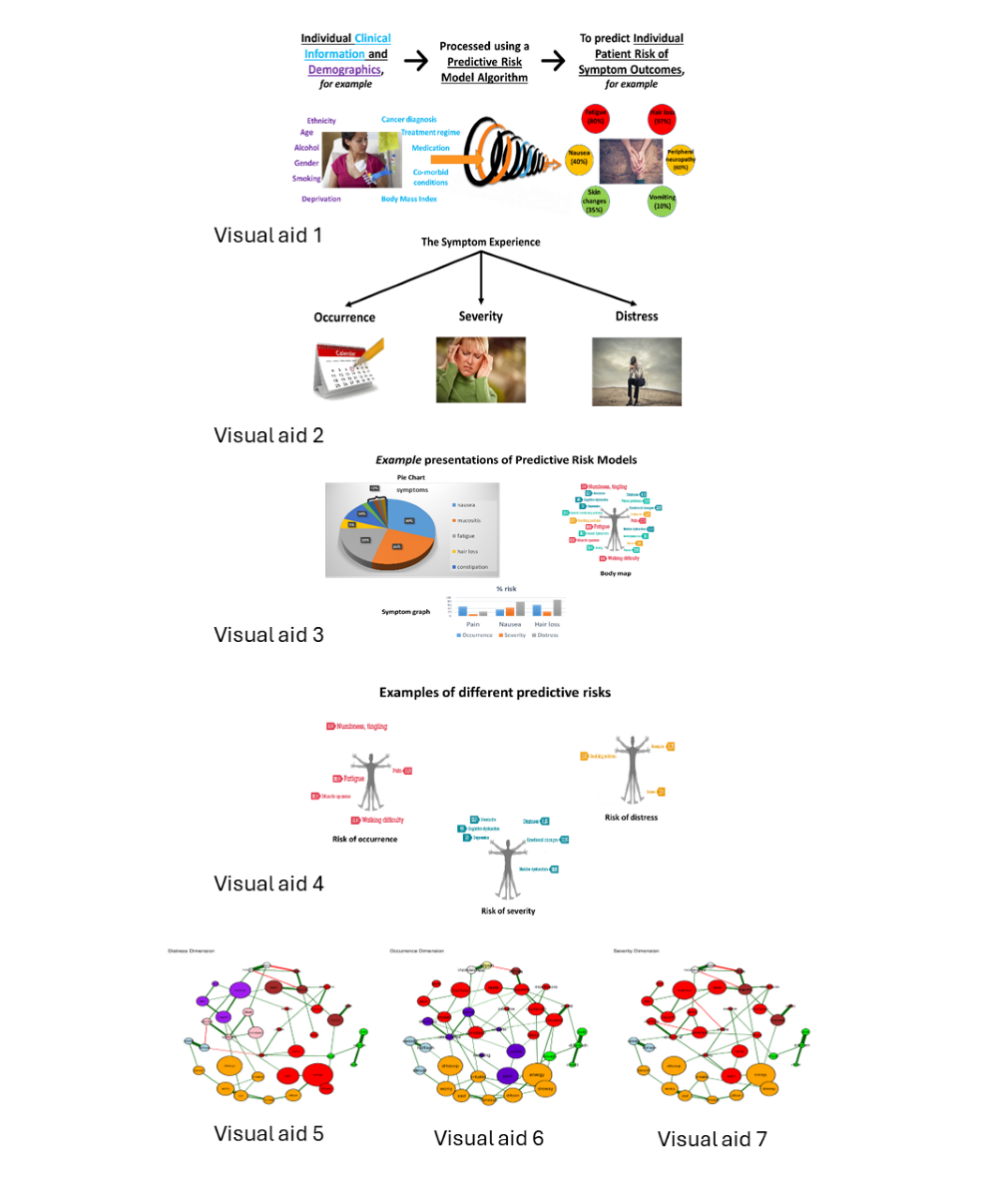
Visual aids used alongside topic guides.

Patient and clinician interviews and focus groups were conducted separately. Interview guides were developed for each sample (patient and clinician) that aimed to explore and understand patients’ and clinicians’ perspectives on the utility and real-world application of the PRMs on CRS. These guides were devised by the study team to ensure that they focused on key questions. While patient and clinician topic guides addressed the same issues and followed the same format, minor changes were made for each target group (Figures 1 and 2 ). Given the novel nature of the concepts under discussion, visual aids were used to facilitate discussions (Figure 3). These visual aids provided examples of ways in which risk predictions of CRS could be presented to support understanding of the concept being discussed and to explore ways in which risks could be visualized and understood by patients and clinicians.

Data were collected between January and April 2019. The main study team provided education and training to each of the clinical sites to conduct the focus groups and interviews in the participants’ native language. The education included concepts to be discussed and the interview guides and visual aids to be used. In addition, the clinical sites received all of the material that would be needed to conduct the interviews or focus groups. Data collection involved 8 interviewers: 5 female and 3 male. Further, 2 of these interviewers were members of the main study team while the remainder were principal or local investigators at the clinical sites. While some interviewers may have been familiar to the clinician participants, they had no direct clinical or line management relationships with them. The interviewers were entirely unknown to the patient participants. For each site, the initial interview or focus group was evaluated by a member of the main study team and feedback was provided to optimize data collection. Except for 1 interview that was conducted by telephone, the other 4 interviews and 10 focus groups were conducted in person.

### Data Synthesis

Interviews and focus groups were recorded on encrypted digital recording devices and uploaded to a secure cloud-based server by all clinical sites. The recordings were transcribed verbatim by a transcription company. For the non-English recordings, the transcriber listened to them in the original language and then transcribed them into English. The correctness of the transcriptions was verified by a member of this study’s team whose native language was German, Greek, or Norwegian who listened to the recordings and read the English translation simultaneously. Any errors were corrected in the transcript before analysis.

### Data Analysis

Qualitative data were analyzed using NVivo software (Lumivero). Thematic analysis, a method of identifying, analyzing, organizing, describing, and reporting themes found within a data set [38], was used to answer this study’s aims. The initial coding framework was based on the patient and clinician interview guides and data from the 2 groups was analyzed together. Common overarching themes were identified in both the patient and clinician data. However, within the subthemes, both convergent and divergent themes emerged between the patients and the clinicians.

Data analysis, coding, and findings were reviewed at weekly team meetings of the lead team of the eSMART RCT to encourage collaborative discussion about the data and facilitate the interpretation of findings. Further, 2 researchers (RM and M Miller) were responsible for completing the thematic analysis individually and then meeting to discuss. A review of the full data set was conducted by a further member of this study’s team (LL) to verify the completed analysis. The main study team concluded that saturation was reached with this sample.

## Results

### Overview

In total, 23 patients participated in 4 focus groups in the United Kingdom, Austria, Norway, and Greece. These focus groups ranged in size from 4 to 6 participants. All 5 patients from Ireland opted for individual interviews due to logistic concerns related to travel, challenges in finding a mutually convenient time, and ongoing symptoms (such as fatigue) following chemotherapy. In total, 26 clinicians participated in 6 focus groups across all 5 countries. These focus groups ranged in size from 2 to 6 participants.

### Participants

Table 1 describes the patients’ and clinicians’ characteristics concerning country, number, age, and diagnosis (patients only). Details on the clinicians’ roles are presented in Table 2.

**Table 1:** Participant demographic characteristics.

	Patient sample										Clinician sample		
	Breast cancer			Colorectal cancer			Hematological cancer			n			Mean (SD)
	n	Mean (SD)	n		Mean (SD)	n		Mean (SD)					
United Kingdom (3 sites)	5	51 (10.7)	3		57 (5.7)	—^a^		—	8			41 (10)	
Ireland (1 site)	4	51 (15.8)	1		70	—		—	6			—	
Greece (1 site)	1	42	1		70	2		69 (8.5)	5			41 (10.1)	
Austria (1 site)	4	55 (11.3)	—		—	2		56 (3.5)	4			43 (7.9)	
Norway (1 site)	4	53 (4.2)	1		31	—		—	3			—	

^a^—: not available.

**Table 2:** Clinician roles.

Roles	Participants, n
Doctor	2
Nurse (staff nurse, oncology nurse, and chemotherapy nurse)	9
Nurse consultant	3
Clinical nurse manager	3
Clinical nurse specialist	4
Head nurse	2
Patient information lead	1
Liaison nurse	2

### Themes Identified

From the data analyses, while overarching themes (ie, perceived benefits of PRMs, negative perceptions of risk, and using risk prediction data) were common across both groups, distinct differences emerged in some of the subthemes by group. Therefore, patients’ and clinicians’ findings are reported separately.

### Patient Themes

Analysis of the patient data identified 3 main themes and several subthemes (Table 3). Specific details for each theme are presented in Table 3.

**Table 3:** Patient themes and subthemes.

Themes	Subthemes
Positive perceptions of predicting risk	Managing expectations Enabling self-care Planning, preparing, and being in control Educating friends and family members
Negative perceptions of predicting risk	Skeptical of concept Negative mindset Self-fulfilling prophecy
Use of risk prediction data	Visualizing the data Frequency of data Preferences for technology

### Positive Perceptions of Predicting Risk

#### Overview

Patients were able to see positive uses for information provided through the use of PRMs for CRS. These uses included helping them to manage symptom expectations; enabling self-care; planning, preparing, being in control; and educating family members and friends.

#### Managing Expectations

Most patients spoke positively about being able to access personalized predictions of symptoms that they were most likely to experience at the start of chemotherapy. They perceived that this information would help them to better manage their expectations of treatment, reduce associated distress, and improve adjustments to treatment and its impact. Further, 1 patient spoke about how having this individualized information could make them feel “a bit more special...”:

Because I would be prepared for [me], not for what chemotherapy does to people as a whole….Treatment cannot have the same symptoms to everyone. I would want the targeted information to be for [me] and not based on a protocol, and the symptoms would be accurate. Just like I know the treatment, I would want to know the symptoms for me. I think that this would be the best.

site 5, patient 4

I was dreading nausea so much because that’s the worst thing I know…[it] would have been really good for me to know that the prognosis shows that I’m likely to not get it – that would have saved me from a lot of dreading.

site 7, patient 3

Patients highlighted how personalized predictions of CRS would help them distinguish between symptoms that were or were not related to their chemotherapy. They perceived that they would be able to distinguish the cause of symptoms and decipher if they were “normal” for them or not. In addition, they suggested that these predictions would have a role in ensuring that patients report “relevant” symptoms to clinicians in a timely manner:

I think it would be easier to handle. Knowing that something is a symptom of something makes it also easier to talk about. Otherwise, it’s like this, ‘I don't know… I feel that… is it normal or not.’

site 6, patient 1

Merely to be able to self-assess my situation and say how significant is this, is this normal? This is what used to bother me the most. I had some side effects, I felt something and the uncertainty of whether I should report it, whether it’s something that needed some specific course of action or whether it was something completely normal.

site 6, patient 6

#### Enabling Self-Care

Patients spoke about how predictive information would allow them to better plan and prepare and enable them to proactively care for themselves:

Okay, I can get some creams in for that and have them ready, rather than going shopping for the stuff...when you—when it happens. So, you have everything under the sun ready to go just in case.

site 1, patient 2

If I’m going to be sore on this day, maybe I could take a pain killer or something that would, that would stop it, like.

site 4, patient 1

#### Planning, Preparing, and Being in Control

A few patients highlighted how PRM information would support them in their daily lives. For example, this information would enable them to work during treatment and manage childcare responsibilities.

To know if I would be able to go to work, if I could get two-three days sick leave, to schedule my life, my daily routine, to take painkillers beforehand.

site 5, patient 4

I think, for me, it would have allowed me to plan a lot better. Because I’m at home with just my son, I’m a single parent. And it would have allowed me to get, or to have more support around me.

site 1, patient 4

#### Educating Friends and Family Members

An interesting perspective was the way that a few patients spoke about how they would share this information to help educate friends and family members. This risk prediction would convey to others what symptoms they, the patient, would likely experience and allow those individuals who were close to them to prepare themselves in advance:

Then the person you’re living with can also be prepared.

site 7, patient 1

It’s never just about you… you’re bringing people with you, partners, family, parents and whatever. And having to explain these are the things I might suffer from, they’re able to see it visually... that might be useful.

site 4, patient 5

### Negative Perceptions of Predicting Risk

#### Overview

In addition to positive perceptions of PRMs, several patients had some negative perceptions of them.

#### Skepticism About the Concept

Some patients were skeptical of the overall concept. They did not believe that it was possible to accurately predict the likelihood of CRS:

It sounds a bit like science fiction that you could say this in advance.

site 6, patient 1

I mean, they’re [PRMs] not going to say you are definitely going to get that [symptom], can they?

site 1, patient 1

#### Negative Mindset

Other patients spoke about how PRM information could be detrimental to their well-being and result in a negative mindset. These patients preferred taking an uninformed approach to CRS:

See that’s the deal, some [people] could be completely negatively affected with this information, and others are not affected at all. And if the probability is that you will be affected by something with an average risk of 90%, of course it’s going to impact your mind negatively.

site 6, patient 2

It could be a worry more than desired, as far as I’m concerned.

site 7, patient 2

#### Self-Fulfilling Prophecy

Linked to these perceptions of PRMs creating a negative mindset, a few patients spoke about predicting risk as a self-fulfilling prophecy. They perceived that being given personalized predictions of likely CRS would affect them psychologically and result in them experiencing these symptoms simply because they were told that they were at increased risk:

Beforehand? I would not want that, because I am easily influenced and I would experience them, even if I was not meant to. From a psychological point of view alone, I would not want that, no.

site 5, patient 2

If you’re told you’re going to be ill on a certain day…you think…oh, I don’t feel good.

site 1, patient 3

### Using the Data From PRMs

#### Visualizing the Data

Visual aids provided examples of how PRM information about CRS could be presented (Figure 1). When asked, patients expressed their preferences for different approaches that could be used to visualize and facilitate their understanding of PRMs. Several patients spoke positively about risk predictions being presented within a cluster of symptoms, seeing the benefit in visualizing how their predicted symptoms “joined up.” They felt that this type of visual aid would help them understand why they experienced certain groups of symptoms and their interdependencies:

I quite like it because it shows that if you get one, you’re more, you know, like the energy, the drowsiness, the, uh, difficulty in sleeping and the fact that they all go together.

site 1, patient 1

It was partially related and depending on one another, because once you have nausea and constipation, you lose your appetite.

site 6, patient 3

Conversely, some patients commented that the cluster visualization was too complicated and better suited for clinicians:

For me, presented in this manner, it just doesn’t help me, it doesn’t make sense.

site 1, patient 4

I mean if this is for the doctors then fine, but I wouldn’t like to have it as a patient.

site 3, patient 4

Some patients suggested that the risk predictions could be presented in a simpler format and proposed a “traffic light” priority system that would enable them to decipher what symptoms were problematic and what ones were not:

I think…the traffic light system is a good system. Because red, we all know that red is danger and not good, so if you have got something in there what you might get it’s something that you will look out for.

site 3, patient 1

#### Frequency of Data

In terms of the timing of the receipt of PRM data, most patients favored receiving information at the start of each cycle of chemotherapy—with a sense that being given daily information would be overwhelming. Having new information with each cycle of chemotherapy was perceived as useful because patients acknowledged that symptom experiences could change over time:

I think, for me, it would be too much for me to handle if I’m getting a……for example, if it’s a text or alert coming through ... today you might vomit [laughter]. Great! Have a nice day [laughter]!

site 1, patient 4

After every treatment there are more symptoms you see. It takes a while for the symptoms to manifest themselves because it is a build-up I suppose… I do think it is a build-up and the tiredness, that’s a build-up as well, at first you’re not too bad and then… but yeah, I would like to know [symptom information] ongoing.

site 4, patient 4

I think having such a prognosis once per month, at the beginning of the month, that would be enough. It should just contain things like, ‘This can happen in this timeframe’. And the second month there is a probability that other symptoms occur. That you have this in advance but not get bombarded with this information all the time.

site 6, patient 3

#### Preferences for Technology

Patients identified the usefulness of having access to the visual representation of risk data on mobile technology (eg, smartphones). They suggested that being able to interact with the data (eg, tap on a symptom to view self-care information) would add value for them:

And I can imagine this to be on a smartphone because everyone has a smartphone anyway. And you see a notification with a new message. And you get this maybe every 2 weeks, 4 weeks, or so. And if this happens, the probability is this and that, take this and that.

site 6, patient 4

I like the idea of being able to either hover over it or tap on a certain thing…and get more information… it’s that positive reinforcement of you’re doing the right thing. So, it’s good to have that little bit of background, that little bit of information that is kind of either telling you that or telling you something a little bit new.

site 1, patient 2

However, despite all of their positive suggestions about the usefulness of PRM information, patients still wanted the reassurance of knowing they could contact a clinician when required:

And I am assuming that the app wouldn’t sort of take away from the fact that there is a person that you can call if you wanted to? Because if it ruined that possibility then it would be a bit worrying I think.

site 3, patient 2

### Clinicians’ Themes

Analysis of the data identified 3 main and several subthemes from the clinicians’ focus groups (Table 4). Each theme is presented in greater depth in Table 4.

**Table 4:** Clinicians’ themes and subthemes.

Themes	Subthemes
Positive perceptions of predicting risk	Anticipatory, preventative, and targeted care “Unique” patient education
Negative perceptions of predicting risk	Trust (in the concept) No additional benefits of artificial intelligence
Use of predictive risk data	Visualizing the data Frequency of data Preferences for technology

### Positive Perceptions of Predicting Risk

#### Overview

Clinicians perceived several ways in which PRMs would positively influence their practice.

#### Anticipatory, Preventative, and Targeted Care

According to the clinicians, one of the main benefits of having PRM information was the way it could promote a more anticipatory and preventative approach to CRS. This approach could decrease patients’ distress and enable them to feel more in control during treatment:

I can recognise problems earlier and intervene earlier, in the sense that I can prevent problems instead of solving them. So I don’t want to wait until the patient has to go the ER on the weekend, but I can take preventive measures in terms of higher dosage and medication management. It’s useful and saves me a lot of work and saves the patient pain and eventually saves the entire medical system costs.

site 6, clinician 1

If the information is there and it’s predicted then you can actually reduce the possibility of unpleasant experiences for patients. Because once they experience one unpleasant experience then the subsequent treatment will become quite intolerable for them, because they anticipate the undesirable reaction.

site 2, clinician 2

In addition, clinicians suggested that PRM information would enable them to better target and manage the side effects of treatment, optimize patient outcomes, and create efficiencies in services:

Knowing the severity levels and the burdening levels with individual patients, and what the stress is, so these three components, it is quite targeted. Because if I take any action, I can have the most beneficial effect, so on one side it is a very efficient measure, and on the other side, it has the advantage that I can prove better that it really is useful. And with this it is easier to divide the resources, and can save resources to be able to target care to this certain patient group.

site 6, clinician 3

For example, if we knew ahead of time that they [the patient] was at a higher risk for permanent hair loss then we might say encourage scalp cooling before they start, in case the scalp cooling helps save what hair they have….because, we have had some ladies in the past who have had permanent hair loss and they’ve said that had they known ahead of time they would never have chosen the adjuvant chemotherapy, because, it [hair loss] restricts their life so much now because of their distress over the symptom.

site 2, clinician 1

#### “Unique” Patient Education

The availability of specific predictions of CRS was perceived by clinicians as an effective way to provide patients with individualized and targeted education about self-care. Clinicians viewed this personalized approach as having the potential to be more effective than general standardized approaches:

This is very important as far as the patient counselling goes. That you don’t have to tell them about all the possible symptoms that can happen, but instead, just thoroughly discuss the ones, three let’s say, that are most likely in his/her case. And just focus on those, and certainly, if some of the others come up, they can get it touch to discuss those. It’s better for the patient because they would go home not with less information but more targeted information.

site 6, clinician 3

I would want that [PRM] information because it would influence the information that I then needed to give them [the patient] to help prepare. Because right now, say in a pre-chemotherapy setting we have to tell them about all the different side effects they might get…But, that means that some patients like in the past have come back and have said that was much worse than I expected or that was not as I expected… You know, because we are gauging it to, not specifically for them.

site 2, clinician 1

### Negative Perceptions of Predicting Risk

#### Overview

Despite their positive perceptions of PRMs, clinicians had some negative comments about the use of PRMs in clinical practice.

#### Trust (in the Concept)

Similar to the patients’ perspectives, some clinicians were skeptical that PRMs would be able to accurately predict individual patient’s symptom experiences. They questioned whether what was proposed was actually possible. Others questioned the evidence base for these types of models and their reliability.

I don’t know until how far a predictive model could go. Until which point can we predict? Until which phase of therapy and patient monitoring we can predict? Can we do that?

site 5, clinician 2

I don’t know if I could see it working

site 4, clinician 4

While recognizing several benefits, a few clinicians raised concerns that these predictions would result in patients having increased anxiety which resulted in more frequent contacts with clinical services.

I think there can sometimes be a certain aspect of…if you’re saying to a patient in their pre-chemo consult this is a 90% likelihood that this is going to make you sick, they’re going to be hypervigilant for that. And, any sign kind of sign of sickness they are going to be straight on the phone and feeling more anxious about it.

site 1, clinician 3

It has happened to me, that a patient, a young one, even though young people usually ask more questions, and she told me that she did not want to know, because she would be expecting the symptoms.

site 5, clinician 6

#### Unable to Deal With all of the Complexity

The ability of PRMs to deal with the complexity of cancer care and the multiple factors that contribute to patients’ symptom experiences was questioned by some clinicians. Some clinicians suggested that the models would not account for human complexity:

Whether it is because of the complexity of the cancer patients, not all of them are well before they start treatment, by the time they start treatment they could be a second line, or third line treatment, they are so unwell. So, my concern would be that those predictors occurrence and so on, would they disregard the general health before the treatment?

site 2, clinician 2

I don’t think you can predict [symptom] distress because it depends upon the person’s coping and ability to cope with what is happening.

site 2, clinician 1

Clinicians doubted that knowledge derived from PRMs could equal the skills of an expert oncology clinician to adequately identify and predict the complex issues associated with caring for patients with cancer:

The only problem with algorithms and AI is that it needs to learn, at the moment it is not complex enough to actually deal with a good chemotherapy nurse. And also, it doesn’t take into account at the moment the patient holistic baselines.

site 1, clinician 3

Several clinicians stated that they already followed anticipatory and preventative models of care provision for symptoms. Therefore, they questioned whether PRMs would add any value to current or future clinical practice:

I think it is used already…in the pre-treatment consultations to target what patients should be aware of, that are most likely to happen in a lot of cases. We use it as well when someone rings up if we know what treatment they’re having, then we can see what is normal.

site 1, clinician 2

We do work preventatively; we do that with many.

site 7, clinician 3

I tend to work with that anyway, with the kind of evidence base on the side effects that we know occur with chemotherapy. And, I suppose from clinical experience then you know that certain patients will have more severe symptoms if that makes sense.

site 1, clinician 1

### Using the Data From PRMs

#### Visualization of the Data

Similar to patients’ perspectives, clinicians were able to see the benefits of presenting the risk predictions within a cluster of related symptoms as an acknowledgment of the relationships between or among specific symptoms.

I think it’s quite useful, it might help with your assessment something like this. Or if somebody rings up with say taste change, kind of helps you to structure the questions so you might ask around it. So for example, is it affecting your appetite, have you lost any weight, is your mouth dry, you know, so it does help with that assessment actually.

site 1, clinician 3

A few of the clinicians liked the way that risk predictions of CRS as a cluster could be presented based on the different dimensions of the symptom experience (eg, severity and distress):

What we see a lot, is the data about the severity [of symptoms] in a lot of scientific work, but about the distress, we actually don’t know very much.

site 4, clinician 2

However, concern was expressed about the challenges of viewing predictions of the risk of chemotherapy symptoms based on their distress alone:

So, it can actually be quite deceiving to see what distresses the patient, because to a patient maybe hair loss is the most important factor, but yet for a healthcare professional, the diarrhoea …they may have actually under-estimated the diarrhoea that could be actually life threatening… they have been having diarrhoea for so long but they didn’t think it was as important as the hair loss.

site 2, clinician 2

Being able to visualize a higher level of risk for a specific symptom within a cluster by the size of its “bubble” (Figure 1) and using the size of the bubbles and links to decipher levels of interdependencies between associated symptoms was viewed as a positive aspect of this approach:

…you would pick the three biggest bubbles if that is all you could address and then they will show you the relationship between the larger bubbles and other things that are influencing it. So, in that sense it is helpful. Right, because you can see what’s maybe influencing the larger bubbles.

site 2, clinician 1

However, concerns were voiced that focusing on symptoms within a cluster that were depicted as having the highest level of risk, may draw attention away from more important or life-threatening symptoms:

So if my attention is being drawn to the things that have the biggest bubble then I am missing the subtle things that are happening that I actually need to pay attention to, because of the type of treatment.

site 2, clinician 1

Clinicians recognized that being able to visualize predictions would be helpful for less experienced clinicians during their interactions with patients:

I think it is useful when you are starting out in a role like this, when you don’t have the experience. I would have found something like this useful, yes.

site 1, clinician 3

I have noticed a lot of new nurses coming on, they quite like models of care. They actually look at temperature and follow the flowchart and not actually looking at the patient. So some [new] staff might actually like this predictive model because it does help them.

site 2, clinician 2

Clinicians recommended that predictive risk information be presented in a simple, visual, way, that patients could easily use and that was intuitive to understand:

So visualisation to quickly see what it is. Visual graphic information goes very quickly into the brain, so if we could have a ‘flash’ graphic display, that would be cool.

site 6, clinician 1

I think it would be good if you could see the whole cycle and then be able to like narrow down on each day. Then you could see change in symptoms, those sort of things or measure against it because they might experience more and more severe at this stage.

site 1, clinician 3

Traffic light system because it is more perceptual, isn’t it? So green is safe, amber is in between, and red is not so good, so it might be something like that.

site 2, clinician 2

#### Frequency of Data

When asked about the frequency of presentations of the predictive risk information, clinicians stated that this information would be most helpful if it were tailored and provided at each cycle of chemotherapy:

I think it is more important for the patients to know at which days during which cycle the symptoms can occur. For example, the first 5-7 days fatigue, such things, so the patient can prepare himself, and orientate themselves towards it. That would be most beneficial to the patients.

site 6, clinician 3

#### Preferences for Technology

Interactivity and incorporation of PRMs into technology was identified by some clinicians as a potential way to integrate this information in practice:

We are in the digital age now... Then you can have the information where you just click, like a tailored bit, this is day two there is a symptom you’re highly likely have that.

site 1, clinician 4

The more graphic the better, a graphic overview, a running chart and having distinct indicators for on-edge situations. Having an alarm-like indicator and a pre-alarm indicator, so to speak.

site 6, clinician 2

## Discussion

### Principal Results

The focus groups and interviews with patients with cancer and clinicians across 5 European countries generated valuable and novel insights into their perceptions about the utility and usefulness of CRS predictions in daily life and clinical practice. Patients and clinicians commented on the practical and clinical advantages of using PRMs to prevent and manage CRS. However, with some reservations about the validity of PRMs, they highlighted that how this information was presented would impact its usefulness and usability in clinical practice. Both groups suggested alternative ideas for how to visualize PRM information. They emphasized the importance of simple and user-friendly formats to maximize benefits. These types of explorations of data visualization and associated usefulness and usability of PRM information for CRS management warrant additional investigation. Given that no differences in perceptions, positive or negative, were evident among patients, clinicians, clinical sites, and countries suggest that perceptions about the use of PRMs for CRS are similar.

### Comparison With Prior Work

#### Patients’ Perspectives

Patients perceived that predictive risk information would help them manage expectations, enable them to proactively plan, prepare, and engage in self-care activities, feel in control, and educate family members and friends about the symptoms they were most likely to experience. These findings are consistent with studies that reported positive perceptions of the use of approaches such as machine learning in health care [39]. Providing patients with accurate information that allows them to anticipate symptoms, and engage in coping strategies to minimize their impact, has the potential to reduce patients’ distress [40] and experiences of anxiety and depression [41] as well as increase their satisfaction with information [42]. In addition, increasing patients’ confidence in performing self-care improves their QoL during chemotherapy [43]. Therefore, the provision of individualized predictive risk information about likely symptoms to patients is likely to improve a range of patient outcomes and experiences. However, this approach may not be useful for all patients.

Some patients were skeptical that the PRMs would be able to accurately predict their symptoms. In addition, some commented that these predictions would negatively impact on their lives. Considering the high levels of anxiety and depression that some patients experience [44], clinicians need to assess whether patients want this type of information during their treatment [45]. Patient-centered communication is linked to greater patient satisfaction and needs to be tailored to individual patient’s needs and preferences [46,47]. Clinicians need to work with patients to explain the potential benefits of PRMs and determine their preference for using this type of information. Given that a trusting relationship between patients and clinicians facilitates communication and health care decision-making, reduces patients’ fears, and results in increased adherence to treatment [48], a shift in trust toward technology may be required. Patients may be more likely to view PRMs positively if they feel that their clinicians also do so.

#### Clinicians’ Perspectives

Clinicians were positive about the information derived from PRMs of CRS, particularly in the delivery of anticipatory, preventative, and targeted cancer care. They appreciated the way that they would be able to use this information to provide truly personalized patient education. Given that the burden of cancer care will increase, the ability to predict, identify, and target patients who are at increased risk of a higher symptom burden will assist with the allocation of health care resources. The oncology specialty is facing several concurrent challenges including an affordability crisis [49] and challenges from the COVID-19 pandemic (eg, delayed and altered treatment pathways and reductions in specialist reviews) that are associated with poorer patient outcomes [50]. Given these growing pressures on various health care systems, the cancer community needs to work collaboratively to find ways to mitigate the potential negative impacts and make use of the best available resources to deliver the highest quality cancer care. The use of PRMs of CRS to plan patients’ care and support during chemotherapy provides 1 way to facilitate the most appropriate allocation of resources.

However, as with some patients, some clinicians expressed concerns about the ability of PRMs to accurately predict symptom experiences. Trust is a crucial factor that influences human beings’ interactions with technology [25]. For digital health care technologies to be accepted as trustworthy and evidence-based, they should be based on robust, resilient, reliable, and effective systems [51]. However, given that a lack of trust in technology by both patients and clinicians continues, additional research on the human factors of technology is required [52]. While providing promising opportunities to support clinicians to achieve the best outcomes for patients, methods to ensure that clinicians view predictive risks for CRS as trusted reliable sources of information warrant additional research and education. Patients and clinicians need to be able to trust that the technologies approved for use in health care systems meet robust standards [51,53]. Consequently, a need exists to apply a framework of trust in health-related machine learning based on sound research and conceptual rigor to ensure that developments, such as risk prediction, will be accepted and implemented in clinical practice [54].

Some clinicians questioned whether PRMs provided any additional information. They expressed that, as expert clinicians, they were able to predict symptoms and initiate preventative and timely interventions. An attitudinal shift is necessary, acknowledging the opportunities offered through health-related technology, that can be used to augment clinical expertise for the benefit of all those who provide or deliver health care. For example, endorsement by a trusted authority like the National Institute for Health and Care Excellence, may facilitate acceptance of PRMs by clinicians [53].

### Future Implications—the Importance of Data Visualization

In this study, patients valued the potential of visualizing how symptoms linked with each other. This visualization helped them understand why certain symptoms occurred together. In addition, for clinicians, the visual clustering of symptoms was perceived to be helpful for their assessment processes. However, participants noted that the visual examples of PRM information that they viewed during focus groups and interviews needed to be revised and made less complex. Given that information may influence patients’ decisions and outcomes, clinicians need to use representations that increase patients’ insights and turn data into something meaningful, relevant, and useful. This approach can be a platform for informed discussions about the significance and burden of risks and the implications for an individual and family members [55,56].

The optimization of visualizations of PRMs for CRS is key because it will improve patients’ and clinicians’ understanding of this information and allow them to see symptom patterns and relationships between or among symptoms more clearly. In addition, this increased understanding will enhance their decision-making abilities about symptoms and what proactive measures to take to manage symptoms in a timely fashion. Optimization of visualizations and understanding of predictive risks have the potential to improve patients’ engagement in their care and facilitate communication and collaboration between patients and clinicians.

Sharing complex health-related information in a way that ensures that users can understand and act on it is a challenge in the field of digital health data [42]. Data visualization, and in particular data visualization that patients can easily use, is a more effective approach to communicating key messages and increasing patient and clinician understanding and engagement [57,58]. Indeed, concerning the PRMs for CRS evaluated in this study, patients’ and clinicians’ preferences for the way forward included visual, usable, *and* informative formats. The development of these types of usable and useful visualizations of CRS has the potential to result in profound and positive changes in patients’ experiences, as well as the provision of individualized supportive care delivery. Future work needs to co-design the presentation of PRM information with clinicians, patients, and family caregivers to ensure that the way that information is presented is engaging, meaningful, usable, and useful for all of the intended recipients [59].

### Strengths and Limitations

This study was conducted across 5 European countries. The consistent positive and negative perspectives of patients and clinicians from different countries who have different cultural backgrounds and lived experiences strengthen the generalizability of these findings. That said, it is important to note that the findings may not apply to countries outside of Europe or to different cancer diagnoses and treatments.

Another strength of this study is the fact that patients’ perceptions of the usability and usefulness of PRMs were grounded in real-life experiences as this study recruited patients who had received chemotherapy. While their comments may have been influenced by the severity of their previous symptom experiences, they could draw on their personal knowledge to visualize ways in which this type of information would have an impact on them (both positively and negatively) during their chemotherapy treatment, validating this study’s results.

The decision on whether to use visual prompts during the focus groups was carefully considered in case so doing would influence participants’ suggestions about ways in which this information could be presented. Some of the visual aids were necessary because they depicted real data, while other images facilitated discussion about novel concepts. The individuals who conducted the focus groups were advised to “hold off” on showing the visual aids if conversations and ideas were flowing well and to use them “when necessary” to explain novel concepts. Given that all researchers opted to use all of the visual aids suggests that they were useful to facilitate meaningful discussions.

A limitation of this study is the lack of information about clinicians’ level and duration of experience with chemotherapy services. However, given that the roles of these participants (eg, chemotherapy nurse specialist, nurse consultant, and head nurse) reflect a level of seniority, it is possible that the interviewees were highly skilled and experienced in chemotherapy treatment and care. These clinicians may have different perspectives about PRMs compared to less experienced colleagues. Comparing perspectives about PRMs among clinicians with different levels of clinical experience may provide new and valuable insights.

Another limitation is that the data were not gathered in a way that allowed for subgroup analysis. Perceptions may be influenced by other factors (eg, health or technical literacy) that were not assessed in this study. However, participants provided positive and negative comments about PRMs. Future research needs to explore potential differences in perceptions held by various subgroups.

The inclusion of family caregivers in future research may provide additional insights about the use of PRMs. In this study, patients identified that a potential benefit of PRMs would be to help them explain their expected symptom experiences to family members and friends. Enhanced understanding of symptom patterns using technology has empowered family members to plan and maintain their own lives during their relative’s chemotherapy [60].

### Conclusions

This study provides novel insights into the perceptions of patients and clinicians about the clinical usefulness and usability of PRMs in the management of CRS. While the potential benefits of PRMs for CRS were recognized, additional strategies to increase users’ confidence and trust in the ability of PRMs to support tailored and predictive interventions and care need to be developed and implemented. The presentation of PRM information in usable, useful, and engaging ways would be a way to facilitate implementation, optimize its uptake, and make it meaningful, relevant, and useful to patients and clinicians as well as family caregivers.

Additional research that is focused on building and validating the processes to develop PRMs is essential. However, advances in the analytical processes must be matched with how this knowledge can be integrated into clinical practice. While incorporating the use of PRMs to provide person-centered, effective management of CRS is important, clinicians must maintain the human side of care that includes the fundamental values of empathy, compassion, and trust [61].

## Abbreviations

AI: artificial intelligence
CRS: chemotherapy-related symptoms
eSMART: Electronic Symptom Management Using the Advanced Symptom Management System
PRM: predictive risk modeling
QoL: quality of life
RCT: randomized controlled trial
